# Patients’ experience with MRI-guided in-bore biopsy versus TRUS-guided biopsy in prostate cancer: a pilot study

**DOI:** 10.3332/ecancer.2020.1127

**Published:** 2020-10-20

**Authors:** Silvia Francesca Maria Pizzoli, Giulia Marton, Paola Pricolo, Serena Oliveri, Paul Summers, Giuseppe Petralia, Gabriella Pravettoni

**Affiliations:** 1 Applied Research Division for Cognitive and Psychological Science, IEO European Institute of Oncology IRCCS, Milan 20132, Italy; 2 Department of Oncology and Hemato-Oncology, University of Milan, Italy; 3 Division of Radiology, IEO European Institute of Oncology IRCCS, Milan 20132, Italy; 4 Precision Imaging and Research Unit—Department of Medical Imaging and Radiation Sciences, IEO European Institute of Oncology IRCCS, Milan 20132, Italy

**Keywords:** in-bore MRI-guided biopsy, prostate cancer, patients’ experience

## Abstract

**Background:**

Ultrasound-guided magnetic resonance imaging (MRI)-fusion biopsy and in-bore MRI-guided biopsy (MRGB) have improved the diagnostic pathway in patients with suspected prostate cancer compared to the traditional random sampling of the prostate gland under transrectal ultrasound guidance (TRUS-Bx). The aim of our study was to assess the psychological experiences of patients undergoing MRGB and TRUS-Bx.

**Method:**

Participants completed an *ad hoc* set of 11 items to be rated from 0 (not at all) to 10 (very much) on visual analogue scales and one open question on the most worrisome aspect of the procedure. The set of items evaluated satisfaction with the information received and the possibility to ask questions to the staff; the tolerability of the irritation, duration and discomfort associated with the exam; their level of worry or calm before the exam; the perceived need to undergo the exam; their satisfaction with the exam and willingness to repeat it in the future; and acceptability of the exam.

**Results:**

Between May 2018 and June 2019, 47 participants were enrolled on the day of their MRGB; 24 had previously undergone TRUS-Bx. The MRGB was rated with high positive scores on all 11 items. The lowest ratings regarded the duration of the exam (mean = 6.6) and feeling calm (mean = 6.6). Participants were significantly more satisfied with MRGB than TRUS-Bx, rating it as less painful and more comfortable, necessary and tolerable.

**Conclusion:**

These preliminary results indicate that the MRGB is likely to be more tolerable and acceptable to patients than TRUS-Bx.

## Introduction

In Western and Northern Europe, more than 200 men per 100,000 receive a diagnosis of prostate cancer (PCa) every year [1], making it the most common cancer in European men after skin cancer [2]. In addition, PCa is the second most common cause of cancer-related mortality among men in Europe and the USA [3, 4]. This is coupled with a notable psychological impact. Green [5], for instance, found that PCa patients have the worst physical and role function, two important aspects of quality of life.

The introduction of multiparametric magnetic resonance imaging (mpMRI) of the prostate, carried out and reported according standardised practices (currently expressed in the Prostate Imaging Reporting and Data System (PI-RADS) v2.1 [6, 7], has changed the diagnostic pathway in patients with suspected PCa. The utility of MRI before prostate biopsy has been widely demonstrated, thanks to studies such as PROMIS, 4M, MRI-first and the PRECISION trial [8–11]. In particular, mpMRI allows a large number (14%–28%) of biopsies to be avoided and leads to better biopsy diagnoses in positive cases, as evidenced by an increase in the diagnosis of clinically significant PCa and a reduction in overdiagnosis.

The use of MRI has also changed how biopsies are carried out in patients with suspected PCa. The traditional systematic approach, with random sampling of the prostate gland under transrectal ultrasound guidance (TRUS-Bx), is recognised in the literature as failing to diagnose up to 30% of tumours [12]. The sensitivity of mpMRI in identifying lesions of the prostate has led to the development of biopsy techniques that target sampling to the lesion. This may take the form of cognitive or automated fusion of the MRI images in an ultrasound-guided procedure or an in-bore MRI-guided biopsy (MRGB) [13]. Current European Association of Urologists guidelines [14] recommend that in biopsy-naive patients with a suspect lesion, systematic TRUS-Bx be followed by MRGB directed at the MRI-suspicious areas, while in patients with a persisting clinical suspicion of PCa after having undergone a systematic TRUS-Bx with negative results, they suggest carrying out only MRGB targeted to the lesion [14].

Together with greater effectiveness and precision, advanced diagnostic imaging procedures hold the promise of improving patients’ comfort and psychological quietness [15]. Most diagnostic procedures, being of low invasiveness compared to many therapeutic processes, may be expected to lead to little patient distress or discomfort. Nevertheless, it has been shown that patients experience great anxiety, fear of the unknown and fear of pain when awaiting radiological procedures, which are further linked to poor coping with health-related issues, as well as poor adaptation and recovery [16]. Despite being relatively simple procedures, prostate biopsies are not without side effects that may impinge on a patient’s well-being and cause negative experiences from a psychological perspective. The nature and extent of these effects differ according to the technique used for prostate biopsy. In the case of TRUS-Bx, the most common side effect is pain that lasts for the duration of the exam, which is experienced by 90% of patients [17]. Patients undergoing MRGB, on the other hand, report greater discomfort due to the position they must maintain during the procedure [18, 19]. The differences extend to the post-procedural period as well. Kasivisvanathan *et al* [20] found the incidence of complications in the 30 days post-biopsy to be lower following MRGB than TRUS-Bx. Blood in the urine was experienced by 30% of MRGB patients as opposed to 63% of TRUS-Bx patients. Similarly, pain at the site of the exam was experienced by 13% of MRGB patients as opposed to 23% TRUS-Bx patients in the 30 days after the respective procedures [20]. At the moment, however, the preference for one biopsy procedure over another in terms of the patients’ well-being and acceptance by patients has not been investigated.

The aim of our pilot study was to assess the psychological experiences of a cohort of patients who had undergone MRGB and to compare the experience of TRUS-Bx versus MRGB in a subsample of those patients who had undergone TRUS-Bx in a previous diagnostic session.

## Methods

### Patients and procedure

Between May 2018 and June 2019, male patients were enrolled to this study on the date of their MRGB. Inclusion criteria were (a) to be undergoing MRGB for suspected PCa, (b) having previously undergone mpMRI and (c) being over 18 years of age. The study was conducted according the Declaration of Helsinki and all participants signed an informed consent for their voluntary participation.

All patients had undergone an mpMRI of the prostate, either in our institution or in a referring centre, leading to the detection of a suspicious lesion according to PI-RADS v2.1 criteria [7]. All mpMRI examinations with lesions having PI-RADS scores ≥3 were discussed in a multidisciplinary meeting and the decisions for MRGB were taken collectively.

MRGBs were carried out with the patient in a prone position and, on average, lasted for approximately 36 minutes. After application of local anaesthesia, a needle guide was positioned in the rectum and attached to a positioning device (Soteria Medical, Arnhem, The Netherlands). The MR planning images for needle guidance were sent to the workstation running the software used to control the positioning device. When the radiologist identified the target, the computer transferred the movement indications to the pneumatic motors that reoriented the needle guide to point in the direction of the lesion. The needle was then inserted, and a sample was taken. This process was repeated until the radiologist was satisfied that the lesion was adequately sampled (typically three to five samples).

### Measures

Participants compiled a paper-and-pencil *ad hoc* set of items, consisting of 11 items, to be rated on visual analogue scales (VAS) of 10 cm in length to express the level of acceptability (from 0 = not at all to 10 = very much) of several aspects of the prostate biopsy procedure(s) they had undergone.

The set of items contained two sections: one for the rating of MRGB and one for the rating of TRUS-Bx, with the questions on the rating of experience being repeated identically for the two biopsy techniques. All participants were asked to answer the MRGB section, and if they had ever experienced TRUS-Bx, to answer the TRUS-Bx section as well.

Specifically, the set of items covered the extent to which the staff gave information and were open to questions, the tolerability of the irritation, duration and discomfort associated with the exam; the tolerability of the level of worry or calm before the exam; the perceived need to undergo the exam; the satisfaction with the exam and willingness to repeat it in the future; and finally, the overall acceptability of the exam. The VAS had previously been found to be effective in assessing preferences related to imaging techniques [21] and, in the present study, both the sections resulted in having a good internal consistency (Cronbach’s ***α*** of the MRGB part: 0.90; Cronbach’s ***α*** of the TRUS-Bx part: 0.95).

Patients were also requested to indicate which procedure they would prefer to repeat, if needed, and to provide a written response indicating the aspect of the exam that worried them the most (*Were you calm and not worried before the exam? In case you were worried, what was the thing that worried you the most?*). Participants who had undergone only MRGB did not answer the item asking which exam they would prefer to repeat, if needed.

### Statistical analysis

We calculated the descriptive statistics for the responses to each question and assessed the differences in responses regarding the two biopsies (paired *t*-tests and Cohen’s *d* for paired data) limited to the participants who had undergone both procedures. We also computed the fraction of participants who would repeat each procedure, if necessary. All the analyses were conducted with SPSS (version 26, SPSS, Inc., Chicago, IL).

## Results

### Demographic data

Overall, 47 participants were enrolled in the study, with a mean age of 63.8 (*s.d.*: 7.7, range: 52–80) years. Nine participants (19.1%) had a diagnosis of cancer prior to the MRGB. Thirty two (68.1%) of the participants were employed and 15 (31.8%) were retired. Their mean educational level was 14.1 (SD: 3.9, range: 8–25) years.

Twenty three (48.9%) of the participants had undergone MRGB only. Their mean age was 64.3 (*s.d.*: 8.4, range: 52–80) years and their educational level was 14.1 (SD: 3.5, range: 8–19) years; 17 (73.9%) were employed and the remaining 6 (26.1%) were retired.

Twenty four (51.1%) of the participants had undergone TRUS-Bx prior to the MRGB. Their mean age was 63.3 (*s.d.*: 7.2, range: 53–77) years and their educational level was 14.1 (*s.d.*: 4.3, range: 8–25) years; 15 (62.5%) of them were employed and the remaining 9 (37.5 %) were retired.

### Acceptability outcomes

The VAS responses regarding acceptability of TRUS-Bx and MRGB are summarised for all participants in Table 1. For all assessed dimensions, the average scores for TRUS-Bx were intermediate-high (ranging between 4.7 and 7.4), while for MRGB they were high (range: 6.6–9.2) on the VAS. For MRGB, the mean scores were related to the perceived utility of the exam (9.2), as well as the perceived possibility to ask questions (9.0) were close to the maximum of the scale [10]. The dimensions that yielded the lowest mean scores for MRGB were the duration of the exam (6.6) and feeling calm (6.6).

In the qualitative reports on MRGB, 10 (21.3%) of the participants indicated pain and 8 (17%) reported pain as the most worrisome aspect of the procedure. Less frequent responses included discomfort due to the motionless position by five participants (10.6%), duration by two (4.3%) participants and fear of developing an infection after the biopsy by one (2.1%) participant.

Overall, both the quantitative comparison of perceptions of the two techniques and explicit preference showed greater acceptability of MRGB compared to TRUS-Bx. In all of the assessed dimensions, except duration, patients’ perceptions were significantly in favour of MRBG (see Table 2). Of the 24 patients who underwent both procedures, 18 (75%) indicated a preference to repeat MRGB, if necessary, rather than TRUS-Bx.

The sizes of the effect (Cohen’s *d*) of paired differences ranged from 0.1 (small effect) to 0.9 (large effect). The smallest effect (*d* = 0.1) was seen for the acceptability of the duration of the exam, while the effects for the levels of acceptability of the information received, the annoyance, the tolerability of the pain, fear for needles, the willingness to repeat the exam if necessary, the feeling of being calm and the perceived utility and acceptability of the exam were intermediate in size (*d* ranging from 0.4 to 0.7). The largest effects (*d* ranging between 0.8 and 0.9, respectively) were observed for satisfaction with the exam and for the possibility of asking questions to the medical staff.

## Discussion

A recent study by Kasivisvanathan *et al*. [11] compared the long-term side effects of MRGB and TRUS-Bx a month after the prostate biopsy. Overall, they found that TRUS-Bx patients encountered more side effects than those who underwent MRGB, but psychological aspects related to the experience of the biopsy techniques were not assessed. The present study represents a first attempt to assess the emotional and psychological experiences of patients with different types of prostate biopsies.

We found MRGB to be seen positively relative to TRUS-Bx, with higher scores on all the assessed dimensions of acceptability, except duration of the examination. With MRGB being perceived as significantly more acceptable by the patients than TRUS-Bx, there was a clear preference in the case of facing a repeat biopsy, with 75% of the participants preferring MRGB (‘*If necessary, which one of the two exams would you prefer to repeat?*’).

It is worth noting that, from a qualitative point of view, the mean VAS scores for MRGB were close to the maximum [10] of the VAS for most dimensions of acceptability. Thus, ratings for the acceptability dimensions were not only in favour of MRGB compared to TRUS-Bx, but they were also high on the scale of measure.

The largest effect of the difference between TRUS-Bx and MRGB was seen for the possibility of asking questions to the physician and for the feeling of being satisfied with the exam, with participants expressing the greatest difference in perception, in favour of the MRGB, on these two aspects. Both the tolerability of the pain and the willingness to repeat the exam if necessary showed relevant effects, while the tolerability of the duration of the exam was perceived as slightly different between the two biopsies, with a non-significant difference, in favour of the MRGB.

Although our results were in favour of the MRGB overall, the findings should be interpreted with some care, in light of the limitations that affected the present study. First, we had a small sample, which composed mostly of participants who did not have a prior diagnosis of cancer. Our results should therefore be generalised with caution, considering the dimension of the sample and the fact that patients with prior cancer diagnoses might display different preferences. Furthermore, the small sample size and the chosen items might have shaped the variability within items scores and the resulting analysis. In the responses for TRUS-Bx, all items had a wide range of scores (from 0 or 1 to 10).

This was also true for some items in the MRGB assessment, but the range for other items lower, at roughly half the scale (between 4.7 and 5.5 and 10). Given that the TRUS-Bx procedures were carried out first in all patients, the larger ranges in the responses to TRUS-Bx could represent a recall bias or reflect patients’ greater familiarity with prostate biopsies at the time of the MRGB. The variability could, however, also be associated with the choice of the psychological dimension assessed by the items or with the small size of the subgroup of our cohort who underwent both procedures. A more advanced study design, with an enlarged sample size, might provide insight into the relevant psychological variables.

Second, we cannot rule out the possibility that the level of received information influenced other dimensions. Indeed, MRGB received significantly higher scores on the items related to information received and the possibility of asking questions. Thus, we cannot exclude the possibility that being more satisfied with information and communication influenced participant perception in respect to other dimensions of the acceptability of MRGB. Moreover, patient experiences and perceptions are likely to be heavily determined by local practices and the specific clinician carrying out the respective procedures. In this study, different clinical teams carried out TRUS-Bx and MRGB and steps were not taken to ensure homogeneity between the teams in their approach to provision of information to the patients. Also, to be recognised is that all those who underwent both procedures had undergone the TRUS-Bx first. The familiarity obtained with prostate biopsy in this first procedure may have influenced their level of knowledge and perceptions regarding MRGB. Lastly, we did not assess the participants’ psychological state before the procedures. Consequently, we were unable to exclude differences in the participants’ initial status or expectancies regarding the biopsy as influences on our findings. Future research could seek to expand our knowledge regarding this point. The presence of distress, as well as the levels of trait anxiety [22], and other individual traits (personality, dispositional optimism) might help to shed light on individual perception of the acceptability of the biopsy.

This pilot study provides evidence of distinct psychological experiences between two variants of a relatively simple, invasive diagnostic procedure—prostate biopsy. Moreover, the differences seen in patients’ preference regarding both items pertaining to patient, medical staff interaction and to the procedures themselves, highlight the potential for targeting the acceptability of diagnostic, and not just therapeutic procedures, by giving weight to each patient’s experience within the cancer care pathway. Taking into account patients’ preferences increases patients’ satisfaction with the healthcare pathway [23] and during a delicate process, such as prostate biopsy, being able to increase the satisfaction of the patients could have a beneficial effect not only on the patient itself but also on the physicians and the healthcare system. This may involve adapting clinical practice to consider a patient’s psychological status before the diagnostic procedure, along with relevant individual and interaction dimensions (e.g., patient–physician communication) that may affect the subjective experiences with the procedures [24–26].

## Conclusion

We found MRGB to be more acceptable by patients than TRUS-Bx, and most patients would prefer to repeat MRGB than TRUS-Bx. Future studies and interventions are needed to take into account patients’ experience, preferences and psychological reactions related to diagnostic and therapeutic procedures for cancer, in order to humanise care and improve patient–physician interactions [27].

## Funding

This work was partially supported by the Italian Ministry of Health with Ricerca Corrente and 5 × 1,000 funds.

## Conflicts of interest

Dr. Summers indicates that he is part owner of QMRI Tech, a research services and medical physics support company, which is not associated with the present study.

## List of abbreviations

PCa: Prostate cancer mpMRI Multiparametric MRI
TRUS-Bx: Ultrasound-guided biopsy
MRGB: In-bore MRI-guided biopsy

## Figures and Tables

**Table 1.** Descriptive statistics of the evaluation of the experience with TRUS-Bx (*N* = 24) and MRGB (*N* = 47).

Questions	VAS responses							
	TRUS-Bx				MRGB			
	Min	Max	Mean	SD	Min	Max	Mean	SD
The information I received from the medical staff were satisfying	0	10.0	7.5	2.9	4.9	10.0	8.9	1.3
The physician allowed me to ask questions and was clear in the explanations	0	10.0	7.4	3.0	5.5	10.0	9.0	1.2
Was the annoyance bearable?	0	10.0	5.1	2.8	0	10.0	6.8	2.7
Was the duration of the exams tolerable?	0	10.0	5.7	2.7	0	10.0	6.6	2.7
Was the pain tolerable?	0	10.0	5.4	3.1	5.1	10.0	8.2	1.5
I feel calm when I face needles and injections	0	10.0	6.5	3.4	0	10.0	7.5	2.8
I would be willing to repeat the exam, if necessary	0	10.0	5.0	3.6	1.3	10.0	7.6	2.5
I think that the exam I undergo was necessary	1	10.0	7.3	2.9	5.2	10.0	9.2	0.9
I was calm and not worried before the exam	1	10.0	5.4	3.0	0	10.0	6.6	3.0
Do you feel satisfied with the exam?	1	10.0	5.9	2.9	4.7	10.0	8.7	1.3
Was the examination acceptable? (In terms of distress, duration, pain, etc.)	0	10.0	5.4	3.1	1.4	10.0	7.9	2.1

**Table 2.** Paired *t*-test analysis.

Questions	VAS Responses Mean (SD)		Comparison (TRUS-Bx – MRGB)		Effect size
	TRUS - Bx	MRGB	*t*	*p*-value	Cohen’s *d*
The information I received from the medical staff were satisfying	7.5 (2.9)	9.0 (1.2)	*t* (22) = −2.6	0.015*	0.6
The physician allowed me to ask questions and was clear in the explanations	7.4 (3.0)	9.0 (1.3)	*t* (19) = −2.5	0.02*	0.9
Was the annoyance bearable?	5.1 (2.8)	7.2 (2.8)	*t* (20) = −2.5	0.02*	0.5
Was the duration of the exams tolerable?	5.7 (2.7)	6.6 (2.9)	*t* (19) = −1.0	0.32	0.1
Was the pain tolerable?	5.4 (3.0)	8.5 (1.3)	*t* (20) = −4.3	0.001**	0.7
I feel calm when I face needles and injections	6.5 (3.3)	7.7 (2.5)	*t* (20) = −2.7	0.01*	0.6
I would be willing to repeat the exam, if necessary	5.0 (3.5)	7.9 (2.4)	*t* (20) = −3.5	0.002*	0.7
I think that the exam I undergo was necessary	7.3 (3.0)	9.1 (1.1)	*t* (20) = −3.0	0.007*	0.6
I was calm and not worried before the exam	5.4 (3.0)	6.8 (3.0)	*t* (20) = −2.5	0.02*	0.5
Do you feel satisfied with the exam?	5.9 (2.8)	8.8 (1.2)	*t* (19) = −4.7	0.001**	0.8
Was the examination acceptable? (In terms of distress, duration, pain, etc.)	5.4 (3.1)	7.8 (2.3)	*t* (18) = −2.6	0.02*	0.4

* *p* < 0.05;

** *p*<0.001.
